# Impact of Vegetable Oil Type on the Rheological and Tribological Behavior of Montmorillonite-Based Oleogels

**DOI:** 10.3390/gels8080504

**Published:** 2022-08-13

**Authors:** M. A. Martín-Alfonso, José F. Rubio-Valle, Juan P. Hinestroza, José E. Martín-Alfonso

**Affiliations:** 1 Department of Chemical Engineering and Materials Science, University of Huelva, Chemical Product and Process Technology Research Center (Pro2TecS), 21071 Huelva, Spain; 2 Department of Fiber Science and Apparel Design, Cornell University, Ithaca, NY 14853, USA

**Keywords:** oleogel, vegetable oil, nanoclay, oil structuring, rheology, tribology

## Abstract

We formulated and characterized oleogels based on montmorillonite clay and vegetable oils that could serve as eco-friendly semi-solid lubricants. In particular, we studied the influence of the physical-chemical properties of olive, castor, soybean, linseed, and sunflower oils on the rheological, chemical, thermal, and tribological properties of the semi-solid lubricants. We prepared the oleogels via the highly intensive mixing of vegetable oils with clay at a concentration of 30 wt.%. The oleogels exhibited shear-thinning, thixotropy, structural recovery, and gel-like behavior commonly related to that of a three-dimensional network. The results were corroborated via XRD measurements showing the presence of intercalated nanoclay structures well-dispersed in the vegetable oil. Empirical correlations between the content of saturated (SFAs), unsaturated (UFAs), mono-unsaturated (MUFAs) and poly-unsaturated (PUFAs) fatty acids and the plateau modulus of the aerogels were found. From these experimental results, we can conclude that the fatty acid profile of the vegetable oils exerts an important influence on the rheological and tribological properties of resulting clay and vegetable oil oleogels.

## 1. Introduction

Growing awareness concerning environmental issues and sustainability trends drive the development of new materials based on renewable resources. Many governments and international agencies are promoting the use of alternative raw materials to decrease pollution and waste. A clear example is the gradual replacement of petroleum compounds with natural products [1,2]. Semi-solid lubricants such as greases are generally highly structured colloidal dispersions that possess a two-phase structure consisting of a thickener agent (traditionally metallic soaps or synthetic polymers) and a fluid oil lubricant (generally mineral or synthetic oils). The thickener forms a colloidal network which traps the oil in a micron-sized structure allowing the grease to exist in a semi-solid state. Semi-solid lubricants properties depend on the nature of its components and on the microstructure achieved during their processing [3]. Today, the semi-solid lubricants market is governed by products based on petroleum/mineral oils, and there is a need to promote the replacement of these non-renewable materials by substances obtained from renewable resources. One possible approach is to make semisolid lubricants based on natural nanoclays [4] as thickeners and vegetable oils as fluid lubricants. Among nanoclays, montmorillonite is one of the most interesting due to its low cost and accesability [5]. Montmorillonite consists of inorganic layered silicates which are several hundred nanometers long and have a spacing between layers of just a few nanometers, allowing hundreds of such layered platelets to be stacked into particles or tactoids. Due to their size, nanoclays interact with matter at the atomic, molecular, and macromolecular level [6]. These hydrophobic mineral particles can be also used as fillers or additives for non-polar polymers [7,8]. Clay is relatively cheap, can be produced at industrial scale, and can form unique nanoparticles due to the intercalation of different substances into the interlayer space [9,10]. Several studies have highlighted how the formation of a particle network in multiphase systems, such as dispersions and gels, are strongly correlated to their physicochemical properties [11,12,13]. Several types of nanoclays have been used as thickeners or additives for lubricants to improve the rheological, extreme pressure, anti-wear and friction properties [14,15,16,17,18,19]. Chizhik et al. and Singh and Bhowmick performed studies with nanoclays, such as bentonite and muscovite and observed a significant decrease in both friction and wear [16,17]. Cao et al. [18] investigated the tribological properties of lubricating greases modified with three different types of montmorillonites and showed that inorganic modification can significantly increase the number of electron traps in the base grease, hence leading to improved insulating and anti-wear performance. Recently, Li et al. [19] functionalized graphene/montmorillonite nanosheets, which had remarkable dispersion stability in oil and found that the nanosheets had an outstanding lubricant property when the concentration was 0.4 mg/mL.

Vegetable oils can be considered potential substitutes for mineral oil-based lubricants and synthetic esters [20]. Vegetable oils exhibit a high viscosity index, high lubricity, low volatility, non-toxicity, and biodegradability. The biodegradability of vegetable oils (90–98%) is much higher than that of mineral oil-based lubricants (20–40%) [21]. However, vegetable oils also have drawbacks such as poor thermal oxidative stability, with a tendency for hydrolysis, oxidation, and thermal decomposition [22] as well as a high melting point that restricts their use in some critical applications [23,24]. Since the double bonds in the fatty acid chains in vegetable oils quickly react with oxygen, and a high content of double bonds is found in vegetable oils, such as sunflower, soybean, olive, linseed, and palm. The development of oleogels to formulate eco-friendly semi-solid lubricants based on nanoclay such as sepiolite and organo-montmorillonite with castor and soybean oils have been reported [25,26,27]. Particularly, the effect of the thickener concentration on the rheological, chemical, thermal and tribological properties, of these systems have been studied. The results showed that the content of nanoclay could be used to modulate the viscosity, the linear viscoelastic functions, and the tribological properties of the oleogels. To the best of our knowledge, the impact of the type of vegetable oil on the bulk and surface properties of nanoclay-based oleogels has not yet been studied. The overall objective of this work is to explore the influence of the physical-chemical properties of vegetable oil, such as castor oil (CO), linseed oil (Li), soybean oil (Soy), sunflower oil (SO), and olive oil (OL) on the rheological, chemical, thermal, and tribological properties of montmorillonite-based oleogels. Our results provide new insights on the formulation of effective and renewable semi-solid lubricants.

## 2. Results and Discussion

### 2.1. Rheological Behavior

#### 2.1.1. Linear Viscoelastic Properties

**Figure 1:**
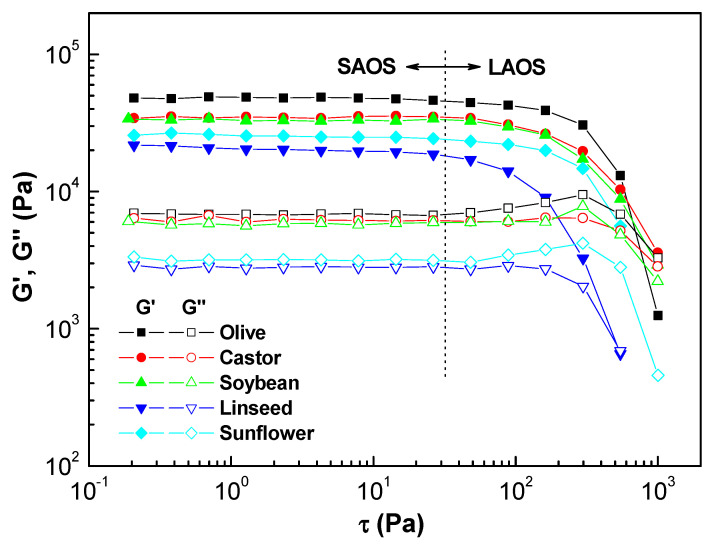
Oscillatory shear stress sweeps for oleogels formulated with different vegetable oils.

To ensure that the frequency sweep tests of the oleogel specimens do not damage the microstructural network, they must be carried out at stress and strain values within the linear viscoelastic range (LVR). Figure 1 displays the results obtained from shear stress sweep tests performed at 1 Hz. The evolution of the storage (G′, filled symbols) and loss (G″, unfilled symbols) moduli were similar to those of soft materials where the application of a shear stress, typically in the order of 0.2–25 Pa evokes a linear elastic response (SAOS) at lower shear stress values followed by a nonlinear response (LAOS) at higher stress values. The decrease of moduli above a critical shear stress (τ**_c_**), indicates a structural breakdown within the gel network. We observed that the loss modulus shows a slight increase at the end of the linear viscoelastic regime, which for some oleogels may be associated with energy dissipation attributed to microstructure reorganization before collapsing [28]. The values of G′ in the LVR reflects the density of interparticle contacts within the network, which were found to be an order of magnitude higher than those for G″. Oleogels formulated with olive, castor, and soybean oils have a high storage modulus, so they are expected to have higher structural rigidity and integrity. The value of critical stress (τ**_c_**) represents the limit of the LVR and it indicates the maximum stress a sample can withstand before the internal structure undergoes permanent deformation. The values of the onset of non-linear viscoelasticity for the oleogels tested are listed in Table 1. Each vegetable oil has a unique interaction with the montmorillonite, so they yielded different microstructural gel networks.

**Figure 2:**
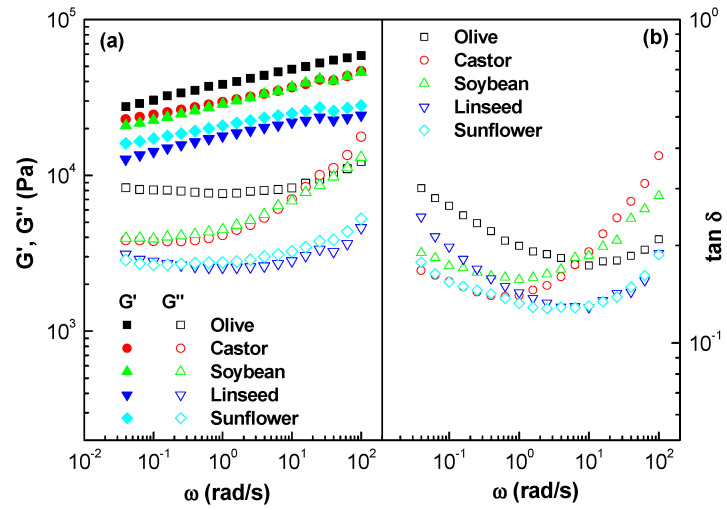
Frequency dependence of the storage and loss moduli (**a**) and loss tangent (**b**), in the linear viscoelastic region, for oleogels formulated with different vegetable oils.

Figure 2a shows the mechanical behavior spectra of oleogels formulated with different vegetable oils. All the spectra exhibited very slow characteristic slopes for the frequency dependence of G′, which is a typical behavior of gel-like materials. In addition, G″ showed a minimum value. While this behavior resembles that of strong gels, the fact that G′ exhibited values just one order of magnitude greater than G″ prevents classifying these oleogels as simple strong gels. We found that the mechanical spectra obtained in our rheological experiments are qualitatively similar to those of a polymer oleogel previously studied [29,30], as well as very similar to those exhibited by some lithium lubricating greases in which G′ values typically ranged from 10^4^ to 10^5^ Pa, at 25–75 °C [31]. The oleogel formulated with olive oil exhibit the highest viscoelastic moduli followed by the oleogels formulated with castor and soybean oil, and the one formulated with linseed oil has the lowest moduli. These results are consistent with the formation of a dispersion of gel-like domains in an oily continuous phase. The presence of a minimum value of G″ is reflected in the occurrence of a further minimum in the log-log plot of the loss tangent (G″/G′) as a function of frequency (Figure 2b). All oleogels showed similar values for the loss tangent. The frequency at which this minimum appears corresponds to a G′ value called the plateau modulus (G_N_^o^) [32]:

(1) $$G_{N}{}^{o}={\left[G^{\prime }\right]}_{\tan\delta \to \mathrm{minimum}}$$

G_N_^o^ defines the so-called rubbery or plateau relaxation zone of the mechanical spectrum. G_N_^o^ and it is associated with the formation of either a transient (e.g., weak gel) or quasi-permanent (e.g., strong gel) network, which is a measure of the density of interparticle interaction in the microstructural network [33].

**Figure 3:**
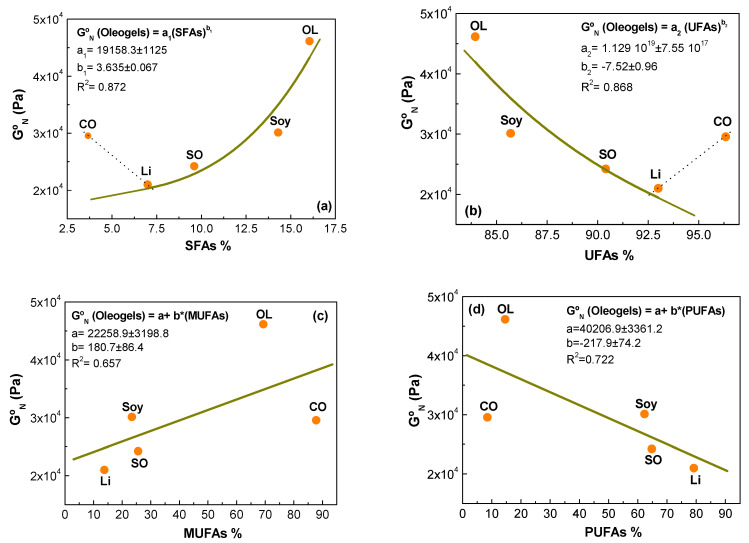
Evolution of plateau modulus of oleogels as a function of: (**a**) Saturated fatty acids (SFAs); (**b**) Unsaturated fatty acids (UFAs); (**c**) Mono-unsaturated fatty acids (MUFAs); (**d**) Poly-unsaturated fatty acids (PUFAs).

Figure 3 displays the influence of the composition of vegetable oils on the linear viscoelastic response of oleogels through the evolution of plateau modulus (G_N_^o^). A correlation is observed between the plateau modulus and the percentage of total saturated (SFAs) and unsaturated (UFAs) fatty acids of the vegetable oils, with the only exception of the castor oil based oleogel. This behavior could be due to the extremely different polarity and viscosity of castor oil. An increase in the G_N_^o^ is observed when the percentage of SFAs increases and when a decrease in the percentage of UFAs is noted. Those trends follow a power-law equation:

(2) $$G_{N}{}^{o}\left(\mathrm{Oleogels}\right)=a_{1}\cdot {\left({\mathrm{SFA}}_{s}\right)}^{b_{1}}$$

(3) $$G_{N}{}^{o}\left(\mathrm{Oleogels}\right)=a_{2}\cdot {\left({\mathrm{UFA}}_{s}\right)}^{b_{2}}$$

where a_1_/a_2_ and b_1_/b_2_ are fitting parameters. In addition, a linear relationship of the content of mono-unsaturated fatty acids (MUFAs) and poly-unsaturated fatty acids (PUFAs) and the plateau modulus is observed (see Figure 3c,d). The linear viscoelastic properties of oleogels depend on several factors such as fatty acids content, polarity, and the viscosity of the vegetable oils used. These factors influence the nanoclay–oil interaction and as a consequence dictate the formation of a weak or a stronger gel network. We found that general vegetable oils with high SFAs/MUFAs content and low UFAs/PUFAs content tend to form stronger microstructural networks.

#### 2.1.2. Viscous Flow Behavior and Thixotropic Properties

**Figure 4:**
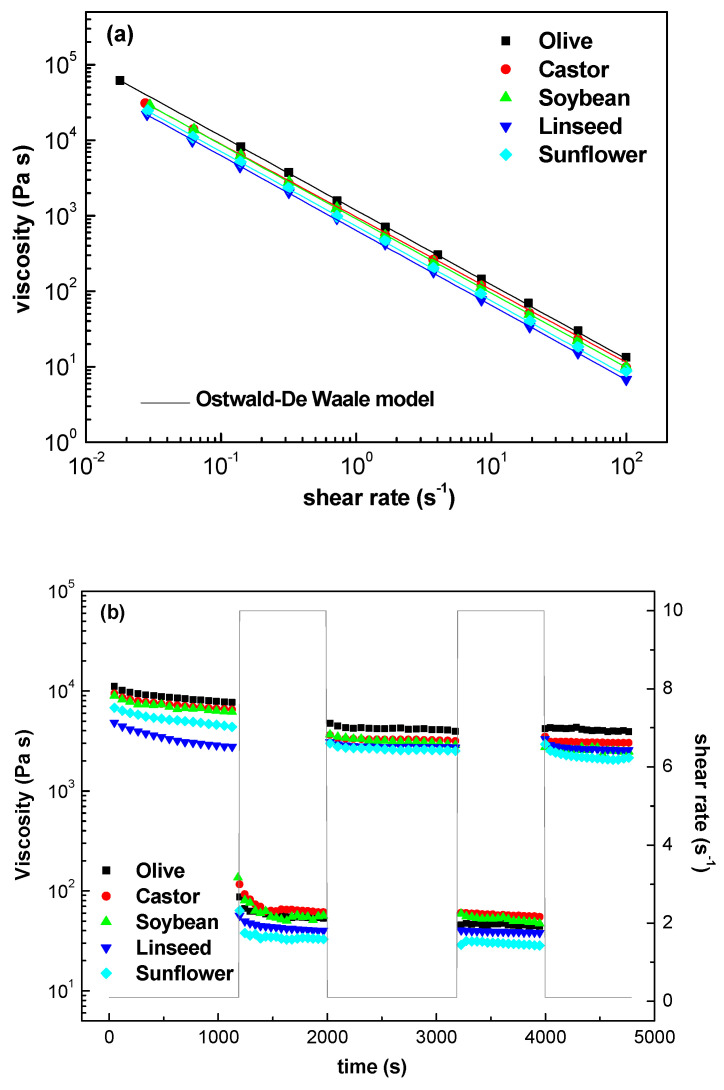
(**a**) Steady shear flow curves as a function of type of vegetable oil. Continuous lines represent fits to the Ostwald-de Waele model and, (**b**) Thixotropic and shear thinning properties of oleogels measured by plotting viscosity over time and at changing shear rates of 0.1 s^−1^ and 10 s^−1^.

Figure 4a displays the viscous flow behavior of oleogels formulated with different vegetable oils using log–log plots of viscosity versus shear rate. All oleogels analyzed exhibited a non-Newtonian shear-thinning behavior characterized by a drop in viscosity with increasing shear rate. We found that the experimental data for all flow curves could be quantitatively fitted to an Ostwald–de Waele model with high (R^2^) values (≥0.990):

(4) $$\eta =K\times {\left(\dot{\gamma }\right)}^{n-1}$$

where η is the viscosity (Pa s), K is a parameter related to the consistency of the sample (Pa s)^n^, $\dot{\gamma }$ is the shear rate (s^−1^), and n is a parameter related to the slope of the shear thinning region, also known as the flow index. The shear thinning properties exhibited by the oleogels are the result of microstructural network disruption followed by an orientation effect as shear increases. The values of the consistency and flow indexes are presented in Table 2. Oleogels formulated with olive, castor and soybean oils, which have the highest monounsaturation and saturation, have higher consistency indexes than the oleogels prepared with others vegetable oils. Oleogels prepared with olive oil presented the highest consistency index value followed by those prepared with castor and soybean oils. Castor oil has the highest monounsaturation, and olive oil has higher saturated fatty acid contents. Meanwhile, olive and soybean oils have similar SFAs values, olive oil has higher MUFAs 69.35% than soybean oil 23.4%. Considering the high melting point of the saturated fatty acids, especially of palmitic acid which is present in olive oil (13.15%), it can be speculated that it could cause aggregation and suspension and that these aggregates do resist the flow [34], hence exhibiting a higher consistency index. On the other hand, the values of flow index, n, were close to zero for all the oleogels studied, which indicates a typical yielding behavior [35]. Yielding behavior is characterized by a decay of several decades in the values of viscosity with a very small increment in shear stress. Thixotropy and shear thinning behavior were also observed for the oleogel samples at low and high shear rates (0.1 and 10 s^−1^). We found that there was initially a progressive decrease in the viscosity of the oleogels at a constant low shear rate over a time period of 20 min (see Figure 4b). When the shear rate was increased, the structure was completely broken down and the viscosity values dropped. However, the structure exhibited some recovery when the shear rate was lowered. These results are of great interest for lubricant applications where a reversible structure breakdown and recovery are desired, and reflect the thixotropic properties and the prominent shear thinning of these oleogels.

#### 2.1.3. Structural Recovery

**Figure 5:**
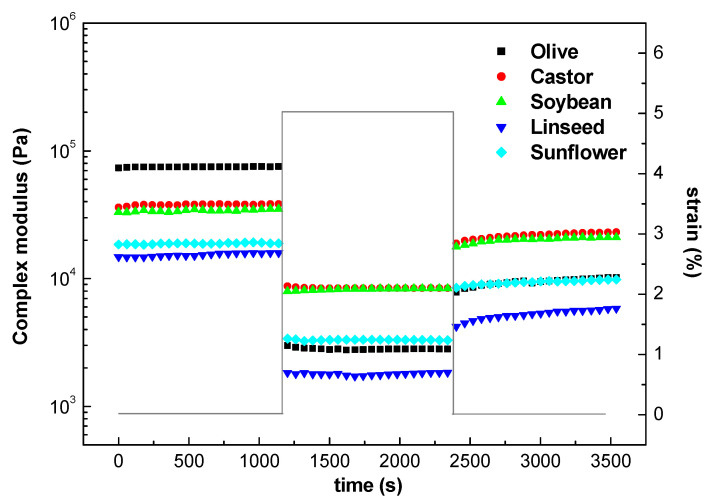
Complex modulus (G*) decay, at frequency of 1 Hz, after changing shear strain from the linear to the nonlinear viscoelastic region and further recovery when the former shear strain is reimposed for the evolution of complex modulus for oleogels formulated with different vegetable oils.

From the oscillatory shear stress sweep experiments, we note that the application of a stress or strain beyond the linear viscoelastic regime results in the destruction of the structure in the specimen being tested. Depending upon the intensity and duration of the imposed stress or strain, the microstructure may either be permanently destroyed or partially recovered. Figure 5 shows the evolution of the complex modulus, G*, a measure of the total resistance of a system towards deformation, for the oleogels when a shear strain outside the linear viscoelastic region is applied and the subsequent recovery when a shear strain inside the linear region is restored. We observed that the oleogels undergo structural breakdown under high strain indicated by a notable drop in complex modulus. When the strain value is restored within the LVR, a marked recovery is seen for most of the formulations. From this plot, it is possible to calculate the percentage of destruction and recovery as:

(5) $$\%\;\mathrm{destruction}=\frac{G_{1}^{*}-G_{2}^{*}}{G_{1}^{*}}\cdot 100$$

(6) $$\%\;\mathrm{recovery}=\frac{G_{3}^{*}-G_{2}^{*}}{G_{1}^{*}-G_{2}^{*}}\cdot 100$$

where $G_{1}^{*}$, $G_{2}^{*}$, $G_{3}^{*}$ are the complex modulus in the LVR (first step shear strain), outside LVR (second step shear strain), and after restoring the stress within LVR (third step shear strain). Table 1 displays the percentages for structure destruction and recovery calculated from Equations (5) and (6). Oleogels formulated with castor and soybean oils show lower destruction percentages, indicating that their microstructures offer higher resistance to structural deformation. On the contrary, oleogels formulated with linseed and especially with olive oil showed a nearly complete structural destruction when a strain outside LVR was applied. Low percentage recovery shown by these oleogels suggests that they were subjected to irreversible structural breakdown. Oleogels formulated with castor and soybean oils showed higher recovery percentages. These results indicate that an oleogel with higher rigidity does not necessarily correspond to one with an increased mechanical stability. For example, the oleogel formulated with olive oil has the highest structural rigidity as reflected by the highest G′, but it also shows an extremely low percentage recovery, suggesting that its structure is sensitive to shear stress. The results from rheodestruction and recovery are of great relevance to the lubricating process. Oleogels are expected to undergo structural breakdown, leading to a decrease in viscosity for ease of lubrication on the surfaces, and when the stress is removed, the oleogels are meant to partially recover to prevent lubricant dripping.

### 2.2. Thermal and Chemical Properties of Oleogel

**Figure 6:**
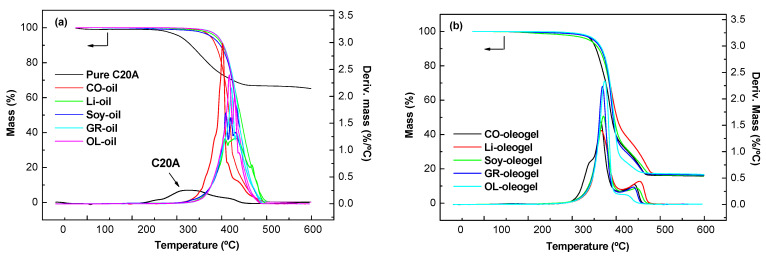
(**a**) TGA/DTG thermograms for pure C20A nanoclay, olive, castor, soybean, linseed and sunflower oils; (**b**) TGA/DTG thermograms for the oleogels formulated with different vegetable oils.

Figure 6a shows the mass loss (TGA) and mass loss rate (DTG) curves for pure C20A organoclay and for the vegetable oils (OL, CO, Soy, Li and SO). The thermal decomposition starts around 300 °C and ends about 500 °C. As for the organoclay C20A, its thermal decomposition shows two stages of mass loss. Firstly, an initial mass loss below 100 °C is attributed to the dehydration of the sample, and a second stage and a shoulder between 180 and 460 °C are attributed to the degradation of the organomodifier (quaternary ammonium salt). Figure 6b shows that the oleogels follow two well-defined stages. The first stage (between 300 and 400 °C) is attributable to the thermal volatilization of the vegetable oil and the second stage (between 400 and 475 °C) may be associated with the degradation of the organomodifier of the nanoclay. Table 2 lists the TGA data, including the temperatures for 5% degradation (T_5_), 10% degradation (T_10_), maximum mass loss (T_max_), and residue (CY) at 600 °C. A 65.8 wt.% residue was measured at 600 °C for C20A which is in agreement with the theoretical presence of an organic ammonium salt. The thermogravimetric data also shows that the content of vegetable oil has a minimal influence on the residue of the stable dispersions. In relation to the temperatures T_5_ and T_10_ of the oleogels and T_max_, there is a difference of ±15 °C, possibly due to the amount of saturated and unsaturated fatty acids. We found that the thermal decomposition behavior of the olegels follow similar trends to those of the vegetable oils with which they were formulated.

**Figure 7:**
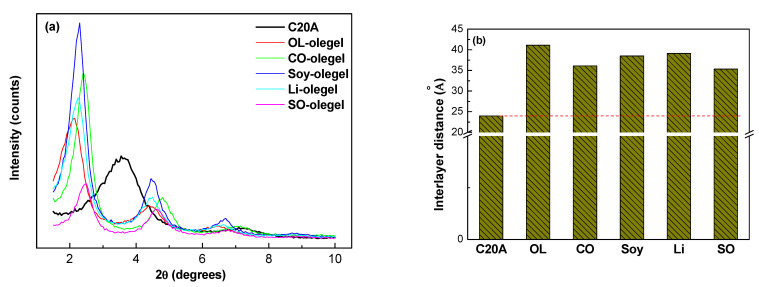
(**a**) X-ray diffractograms for oleogels as a function of vegetable oil. Pure C20A intensity profiles are also included for reference, and (**b**) Interlayer spacing obtained from diffraction peaks on C20A and different oleogels.

X-ray diffraction (XRD) was used to determine the degree of interaction and the extent of intercalation or exfoliation, by measuring the interlayer spacing of the organoclay as it interacts with the vegetable oils. The basal spacings between the structural planes of the clay and the oleogels were calculated from the d_001_ reflection peak in the X-ray diffractogram using Bragg’s equation [36]:

(7) n·λ=2d·sinθ

where λ is the wavelength of the X-ray radiation used in the diffraction experiment, d is the spacing between the planes of the diffraction grating, n is the diffraction order, and θ is the angle of the incident radiation beam with respect to the horizontal plane. XRD patterns of pure C20A organoclay and of the oleogels are shown in Figure 7a. C20A exhibits a main characteristic peak d_001_ at 2θ = 3.65°, which corresponds to the layer spacing of 23.997 Å, together with a small peak at 2θ = 7.22°, which is typical of unmodified montmorillonite [26]. In the XRD patterns of the oleogels, the main diffraction peak characteristic of the organoclay shifts to lower angles corresponding to the interlayer spacings shown in Figure 7b. The larger interlayer spacing observed is related to the affinity between the internal quaternary alkyl ammonium groups in the clay and the oil which facilitates their insertion between the organo-modified silicate layers. Significant differences in the spacing of the layers with respect to C20A indicate that the quaternary alkyl ammonium groups in the clay and the vegetable oils have a strong affinity. X-ray results also confirm that the microstructural network of the oleogels detected in the rheological tests is indeed attributed to the presence of intercalated nanoclay layers. Lower intensity peaks at higher reflection angles, depicted in Figure 7a, could be associated to additional harmonic reflections (002), (003) and (004), as they approximately correspond to multiples of the scattering angle of the first order reflection peaks [37]. The additional reflections might result from the presence of flocculated, intercalated clay platelets with coherent layer stacking, which may remain aggregated or arise as a consequence of a reaggregation process during intensive mixing [38].

### 2.3. Tribological Properties of Oleogels

The influence of vegetable oils on the lubrication performance of the formulated oleogels was analyzed using tribological experiments. Table 3 shows consistency data for all oleogels studied as well as the corresponding NLGI grade. The NLGI grade is a commonly accepted parameter to classify lubricating greases as a function of their consistency degree (between 000 and 6) [39]. The most commonly used greases have a NLGI grade 2. Lubricating greases with soft NLGI grades (0–1) are used in high speed and low load applications at low temperature, while those with higher NLGI grades are suitable for low-speed bearings, operating in higher temperature environments, and required to avoid water washout [40]. Table 3 shows that all oleogels studied showed a NLGI grade around 2. The oleogel formulated with olive oil has a hardness slightly larger, while linseed’s oleogel is lightly softer.

**Figure 8:**
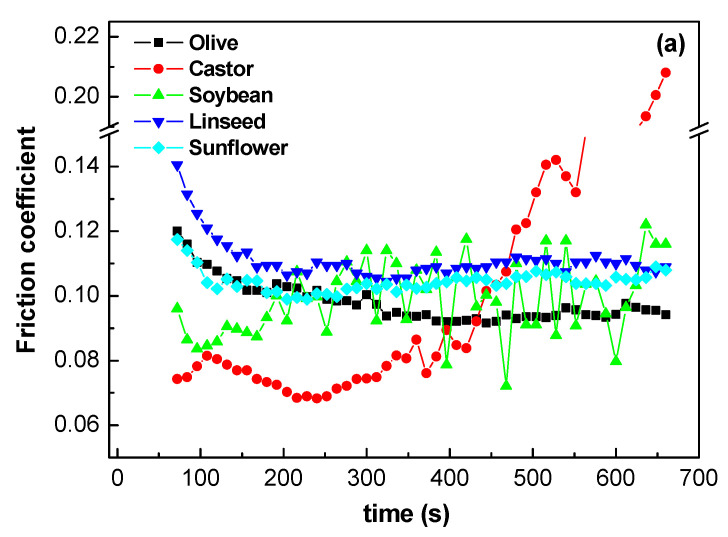

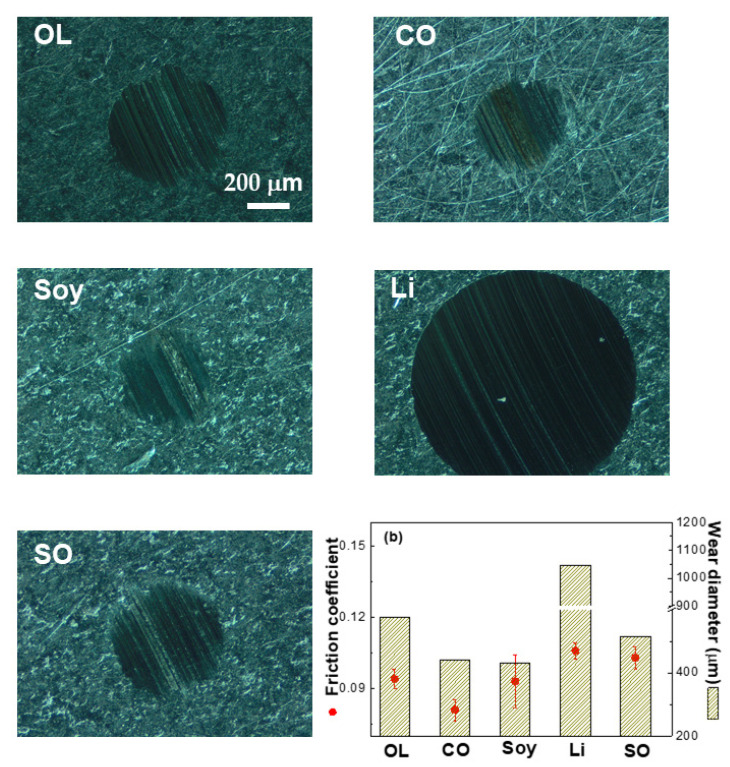
(**a**) Friction curves for oleogels as a function of vegetable oils, and (**b**) Optical images of the worn plate surface and average friction coefficient (points), wear diameter (bars).

The lubrication properties of oleogels were investigated in a ball-on-plate tribological contact device. A specific normal load of 30 N and a rotational speed of 100 rpm were selected to achieve a mixed lubrication regime which was previously determined by performing ramps of rotational speed, i.e., the Stribeck curves. Bearings and moving parts of machines commonly operate in the mixed lubrication region. Mixed lubrication is known to be influenced by the type of oil and additive used, and by the characteristics of the surface of the material being lubricated [41]. Figure 8a displays the evolution of the friction coefficient versus time for all theoleogels studied. Oleogels formulated with olive, linseed, and sunflower oils have a similar behavior, in which the coefficient of friction falls, from a higher value to form a stable sliding as of 350 s, which is indicative of the formation of a protective surface film, promoted by tribochemical processes due to the rubbing action. In contrast, the frictional behavior of the oleogels formulated with castor and soybean oils is characterized by a run-in. Friction coefficients for the oleogels formulated with soybean oil fluctuate with time, a fact that could be associated with lubrication of the contact surfaces, while the friction coefficient of oleogels formulated with castor oil reaches a steady-state value at around 300 s, and then exhibits a sudden increase, which could be attributed to failure in their load-carrying capacity and starved lubrication. The contact surfaces of the steel balls are damaged after the experiments, and the rounded worn surfaces show a rough trace and deep furrows along the sliding direction (see Figure 8b), suggesting that the predominant wear mechanism was abrasion by asperities on the harder surfaces. Figure 8b displays the friction coefficients at 350 s, and the average diameters of the wear scar formed in the steel plates. We found that significant differences in the extent of wear scar depend on the type of oil used to formulate the oleogel. Olive, sunflower, and especially linseed oleogels have higher wear diameters. The values of friction coefficient obtained in the steel–steel ball-on-plate configuration were similar, i.e., 0.093 ± 0.013, to those of a lithium commercial lubricating grease [42].

## 3. Conclusions

We used rheological, thermal, chemical, and tribological experiments to get an insight into the behavior of montmorillonite-based oleogels prepared with vegetable oils. We found that all the oleogels formulated exhibited a gel-like behaviour (G′ > G″) which is typical of structured materials. This behavior was associated to the formation of a well-defined montmorillonite three-dimensional network. The linear viscoelastic material functions were found to depend on the content of fatty acids of the vegetable oils, and empirical linear and power-law correlations with plateau modulus were found to describe well that behavior. Oleogels formulated with vegetable oils containing high SFAs/MUFAs and low UFAs/PUFAs contents achieved higher plateau moduli as a consequence of stronger microstructural networks. All olegels exhibited a thixotropic recovery behavior as well as a strong shear thinning, which quantitatively fits the Ostwald-De Waale model. We also found that oleogels formulated with linseed and olive oil showed poor structure recovery properties. Finally, the differences observed in the tribological studies reflect the strength of the boundary film formed by the oleogels on the surface. Those formed by castor and soybean oleogels were weaker, and thus less able to withstand rubbing. Meanwhile, oleogels formulated with olive, sunflower, and linseed oils had a better frictional behavior. The experimental results show that montmorillonite-based oleogels prepared with several vegetable oils could be used as alternative substitutes to conventional semisolid lubricants formulated with petroleum derivatives.

## 4. Materials and Methods

### 4.1. Materials

A surface modified organo-montmorillonite (Cloisite^®^ 20A) purchased from Southern Clay Products (Texas) was used as thickener agent. Cloisite^®^ 20A is an off white, natural montmorillonite modified with N,N-dimethyl dehydrogenated (C14-C18) quaternary ammonium chloride. The cation exchange capacity for Cloisite^®^ 20A is 92.6 meq/100 g clay and the hydrogenated tallow is composed by a combination of octadecyl (65 wt.%), hexadecyl (30 wt.%) and tetradecyl (5 wt.%) groups. All vegetable oils were used as received. Soybean oil (Soy) and castor oil (CO) were purchased from Guinama (La Pobla de Vallbona, Spain). Olive (OL), sunflower oil (SO), and linseed oil (Li) were acquired from a local supermarket. The fatty acid composition of the vegetable oils was determined by gas chromatography with a flame ionization detector using a previously published method [43]. Table 4 shows the fatty acid composition and main physical properties of the vegetable oils. Oleic (C18:1), linoleic (C18:2 and C18:3), and ricinoleic (C18:1 -OH) acids were the predominant fatty acids (FACs), followed by palmitic (C16:0) and stearic (C18:0) acids. Myristic acid (C14:0) was detected in trace amounts (<1% of total FAs). However, the individual fatty acid (FA) content for each of the vegetable oils tested varied, leading to differences in total saturated fatty acids (SFAs), mono-unsaturated fatty acids (MUFAs) and poly-unsaturated fatty acids (PUFAs). For the vegetable oils analyzed in this work, the percentage of total saturated (SFAs) ranged from 3.66% to 16.05% and that of unsaturated (UFAs) fatty acids from 83.95% to 96.34%. Castor oil has the highest amount of unsaturated fatty acids. The degree of unsaturation of castor oil and of olive oil mainly originates from mono-unsaturation, whereas the unsaturation of linseed and sunflower oils comes from poly-unsaturation. Mono-unsaturated fatty acids, especially oleic acid (C18:1) is the predominant component of olive oil. Castor oil is rich in ricinoleic acid (C18:1: -OH), and sunflower, soybean, and linseed oils are rich in linoleic acid (C18:2) and (C18:3).

### 4.2. Oleogel Formulation

Oleogels were prepared by mixing the organoclay (at a concentration of 30 wt.%.) with vegetable oils, at room temperature, using a rotor-stator turbine (Ultra Turrax T-50, Ika, Staufen, Germany) at 9000 rpm for 8 min. Prior to the high shear processing, the organoclay was wetted with the oil for 60 min at room temperature.

### 4.3. Experimental Methods

#### 4.3.1. Rheological Characterization

Rheological measurements were performed using a controlled-stress rheometer Physica MCR-501 (Anton Paar, North Ryde, Austria) using sand-blasted rough parallel stainless-steel plates of 25 mm in diameter and a gap of 1 mm. We selected a rough surface on account of the risk that the oleogels were prone to slip effects. A rest time of 30 min, introduced before all measurements, was used to relieve residual stresses and to rebuild the microstructure. Rheological tests were carried out to evaluate the rheological behavior of the oleogels: (a) Oscillatory shear stress sweeps at 1 Hz were conducted to estimate the linear viscoelastic range (LVR). (b) Frequency sweeps were used to determine the mechanical spectra under small amplitude oscillatory shear measurements (SAOS) and carried out over the (0.02–100) rad/s frequency range. (c) Steady shear flow properties were determined by multistep flow curves in a controlled-rate mode from 0.02 to 100 s^−1^. To study the thixotropic behavior of the samples, flow curves were obtained by measuring the viscosity of the oleogels at alternating high and low shear rates (0.1 and 10 s^−1^). Finally, (d) dynamic rheodestruction and recovery measurements were performed using triple-step-strain tests in which a strain within an LVR for 20 min was imposed followed by imposing strain outside the LVR for another 20 min. Finally, the strain was restored to the initial value. In order to ensure accurate results, replicates were conducted for every sample/test.

#### 4.3.2. Thermogravimetric Analysis (TGA)

Measurements of mass loss versus temperature were carried out by using a thermogravimetric analyzer, model Q-50 (TA Instruments) under N_2_ purge. Typically, 5–10 mg of sample were placed on a platinum pan and heated from 30 to 600 °C at a rate of 10 °C/min

#### 4.3.3. X-ray Diffraction Analysis (XRD)

X-ray diffraction tests were carried out at 25 °C in a D8 Advance X-ray diffractometer (Bruker-AXS, Germany) with Cu-Ka radiation at a generator voltage of 40 kV, a current of 30 mA and a wavelength of 0.15406 nm. The scan rate was 0.475 min^−1^ in the 2θ range 1.5° and 10°.

#### 4.3.4. Penetration Test and Tribological Characterization

Penetration tests were performed according to the ASTM D 217 standard test method [44]. Tribological tests were performed using a tribological cell coupled to a Physica MCR-501-rheometer (Anton Paar, North Ryde, Austria). The tribology cell has a 1/2 in. diameter steel ball (1.4401 Grade 100, roughness = 0.10 μm) rotating on three 45° inclined steel plates (1.4301, roughness = 0.21 μm). All tests were performed by applying a normal load of 10 and 30 N and constant rotational speed of 100 rpm during 10 min at 25 °C. This test was repeated 5 times to obtain an accurate average friction factor. For each test, a new contact area on the surface of the ball was used. Resulting wear marks in the plates were examined using an Olympus BX52 (Tokyo, Japan) microscope equipped with an Olympus C5050Z camera and a 4× objective lens.

#### 4.3.5. Statistical Analysis

A statistical study was carried out on each of the selected parameters. For this study, an analysis of the variance of a factor (ANOVA) was carried out using two replicates of each measure independently. Then, a series of statistical parameters were calculated, including the mean and the standard deviation. In addition, a mean comparison test was performed to detect significant differences (*p* < 0.05).

## Figures and Tables

**Table 1:** Rheological parameters for oleogels studied.

Samples	τ_c_ (Pa) ^1^	K (Pa∙s^n^) ^2^	n ^3^	Destruction (%)	Recovery (%)
Olive	73.8 ^a^	1179.5 ^A^	0.015 ^aa^	96.3 ^AA^	9.39 ^α^
Castor	80.6 ^b^	964.7 ^B^	0.028 ^bb^	75.7 ^BB^	44.4 ^β^
Soybean	79.4 ^c^	907.9 ^C^	0.018 ^cc^	77.9 ^BB^	46.5 ^β^
Linseed	51.9 ^d^	637.8 ^D^	0.010 ^dd^	88.6 ^CC^	26.9 ^ᵩ^
Sunflower	78.3 ^c^	726.9 ^E^	0.011 ^dd^	82.2 ^DD^	40.1 ^γ^

^1^ Critical stress. ^2^ Consistency index. ^3^ Flow index. Note: Values with different superscripts were significantly different at *p* < 0.05.

**Table 2:** Thermal property data of C20A organoclay, vegetable oils and oleogels formulated with different vegetable oils.

Samples	T_5_ (°C) ^1^	T_10_ (°C) ^2^	T_max_ (°C) ^3^	CY ^4^
C20A	280	307	331/450	65.8
OL-oil	372	385	416/429	0.023
CO-oil	354	367	382/401	0.026
Soy-oil	356	378	409/420	0.031
Li-oil	367	383	408/431	0.028
SO-oil	369	384	421/434	0.038
OL-oleogel	330	346	374/412	16.8
CO-oleogel	321	332	368/402	17.7
Soy-oleogel	316	338	370/445	15.9
Li-oleogel	329	346	365/452	16.3
SO-oleogel	327	344	369/443	16.3

^1^ Temperature at 5 mass % loss. ^2^ Temperature at 10 mass % loss. ^3^ Maximum mass loss temperature. ^4^ CY: char yield, the residue after TGA analysis at a maximum temperature of 600 °C.

**Table 3:** NLGI consistency numbers, penetration and friction coefficient values for oleogels studied.

Oleogel	Penetration Index (dmm)	NLGI Grade	Friction Coefficient
Olive	255 ^a^	2–3	0.096 ^A^
Castor	273 ^b^	2	0.119 ^B^
Soybean	281 ^b^	2	0.101 ^A^
Linseed	302 ^c^	1–2	0.113 ^B^
Sunflower	294 ^d^	2	0.108 ^B^

Note: Values with different superscripts were significantly different at *p* < 0.05.

**Table 4:** Physicochemical properties and fatty acid composition of vegetable oils.

Property	Olive	Castor	Soybean	Linseed	Sunflower
Dynamic viscosity at 40 °C (mPa s)	38.3	230.0	31.4	30.2	31.1
Density at 15 °C (g/cm^3^)	0.9164	0.9630	0.9256	0.9416	0.9216
Myristic ^1^ 14:0 ^2^	0.80	-	0.1	-	-
Palmitic 16:0	13.15	1.70	10.8	4.09	6.19
Stearic 18:0	2.10	1.96	3.40	2.91	3.41
Oleic 18:1	69.35	5.34	23.4	13.8	25.6
Ricinoleic (18:1 -OH)	-	82.48	-	-	-
Linoleic 18:2	14.60	7.01	55.6	14.6	64.8
Linoleic 18:3	-	1.51	6.70	64.6	-
Saturated (SFAs)	16.05	3.66	14.3	7.01	9.60
Monounsaturated (MUFAs)	69.35	87.82	23.4	13.8	25.6
Polyunsaturated (PUFAs)	14.60	8.52	62.3	79.2	64.8
Unsaturated/saturated ratio	5.23	26.32	5.99	13.29	9.42

^1^ Fatty acid concentrations are in % of the total oil. ^2^ C:D, where C is the number of carbon atoms in the fatty acid chain, and D is the number of double bonds.

## Data Availability

Not applicable.

## References

[1] Wu Y., Zhang J., Dong S., Li Y., Slaný M., Chen G. (2022). Use of betaine-based gel and its potential application in enhanced oil recovery. Gels.

[2] Long W., Zhu X., Zhou F., Yan Z., Evelina A., Liu J., Wei Z., Ma L. (2022). Preparation and Hydrogelling Performances of a New Drilling Fluid Filtrate Reducer from Plant Press Slag. Gels.

[3] Martín-Alfonso J.E., López-Beltrán F., Valencia C., Franco J.M. (2018). Effect of an alkali treatment on the development of cellulose pulp-based gel-like dispersions in vegetable oil for use as lubricants. Tribol. Int..

[4] Ray S.S., Bousmina M. (2005). Biodegradable polymers and their layered silicate nanocomposites: In green the 21st century materials word. Prog. Mater. Sci..

[5] Nourmoradi H., Avazpour M., Ghasemian N., Heidari M., Moradnejadi K., Khodarahmi F., Javaheri M., Mohammadi Moghadam F. (2016). Surfactant modified montmorillonite as a low cost adsorbent for 4-chlorophenol: Equilibrium, kinetic and thermodynamic study. J. Taiwan Inst. Chem. Eng..

[6] Saurabh C.K., Gupta S., Bahadur J., Mazumder S., Variyar P.S., Sharma A. (2015). Mechanical and barrier properties of guar gumbased nano-composite films. Carbohydr. Polym..

[7] Uddin F. (2008). Clays, nanoclays, and montmorillonite minerals. Metall. Mater. Trans. A Phys. Metall. Mater. Sci..

[8] Bordes P., Pollet E., Avérous L. (2009). Nano-biocomposites: Biodegradable polyester/nanoclay systems. Prog. Polym. Sci..

[9] Gerasin V.A., Kurenkov V.V., Pashkov O.V., Ilyin S.O. (2017). Structure and rheology of aqueous poly (vinyl acetate) dispersions modified with montmorillonite. Colloid J..

[10] Ilyin S.O., Brantseva T.V., Gorbunova I.Y., Antonov S.V., Korolev Y.M., Kerber M.L. (2015). Epoxy reinforcement with silicate particles: Rheological and adhesive properties—Part I: Characterization of composites with natural and organically modified montmorillonites. Int. J. Adhes. Adhes..

[11] Rehbinder P. (1958). Coagulation and thixotropic structures. Discuss. Faraday Soc..

[12] Zeichner G.R., Schowalter W.R. (1979). Effects of hydrodynamic and colloidal forces on the coagulation of dispersions. J. Colloid Interface Sci..

[13] Varga Z., Swan J.W. (2018). Large scale anisotropies in sheared colloidal gels. J. Rheol..

[14] Gorbacheva S.N., Yarmush Y.M., Ilyin S.O. (2020). Rheology and tribology of ester-based greases with microcrystalline cellulose and organomodified montmorillonite. Tribol. Int..

[15] Martín-Alfonso J.E., Valencia C., Franco J.M. (2014). Composition-property relationship of gel-like dispersions based on organo-bentonite, recycled polypropylene and mineral oil for lubricant purposes. Appl. Clay Sci..

[16] Chizhik P., Dietzel D., Bill S., Schirmeisen A. (2019). Tribological properties of a phyllosilicate based microparticle oil additive. Wear.

[17] Singh H., Bhowmick H. (2019). Influence of nanoclay on the thermophysical properties and lubricity characteristics of mineral oil. Mater. Today Proc..

[18] Cao Z., Xia Y., Xi X. (2017). Nano-montmorillonite-doped lubricating grease exhibiting excellent insulating and tribological properties. Friction.

[19] Li Y., Yang R., Hao Q., Lei W. (2021). Tribological properties of the functionalized graphene/montmorillonite nanosheets as a lubricant additive. Tribol. Lett..

[20] Hwang H.S., Erhan S.Z. (2001). Modification of epoxidized soybean oil for lubricant formulations with improved oxidative stability and low pour point. J. Am. Oil Chem. Soc..

[21] Chauhan P.S., Chhibber V.K. (2013). Non-edible oil as a source of bio-lubricant for industrial applications: A Review. Int. J. Eng. Sci. Innov. Technol..

[22] Pindit K., Thanapimmetha A., Saisriyoot M., Srinopakun P. (2021). Biolubricant basestocks synthesis using 5-step reaction from jatropha oil, soybean oil, and palm fatty acid distillate. Ind. Crops Prod..

[23] Nie J.Y., Shen J.H., Shim Y.Y., Tse T.J., Reaney M.J.T. (2020). Synthesis of trimethylolpropane esters by base-catalyzed transesterification. Eur. J. Lipid Sci. Technol..

[24] Qiao S., Shi Y., Wang X., Lin Z., Jiang Y. (2017). Synthesis of biolubricant trimethylolpropane trioleate and its lubricant base oil properties. Energy Fuels.

[25] Martín-Alfonso J.E., Martín-Alfonso M.J., Franco J.M. (2020). Tunable rheological-tribological performance of “green” gel-like dispersions based on sepiolite and castor oil for lubricant applications. Appl. Clay Sci..

[26] Martín-Alfonso J.E., Martín-Alfonso M.J., Valencia C., Cuberes M.T. (2021). Rheological and tribological approaches as a tool for the development of sustainable lubricating greases based on nano-montmorillonite and castor oil. Friction.

[27] Saxena A., Kumar D., Tandon N. (2021). Development of eco-friendly nano-greases based on vegetable oil: An exploration of the character via structure. Ind. Crops Prod..

[28] Alfonso J.E.M., Valencia C., Valencia C. (2015). Linear and nonlinear viscoelasticity of oleogels based on vegetable oil and ethylene vinyl acetate copolymer/isotactic polypropylene blends. J. Appl. Polym. Sci..

[29] Martín-Alfonso J.E., Franco J.M. (2015). Influence of polymer reprocessing cycles on the microstructure and rheological behavior of polypropylene/mineral oil oleogels. Polym. Test..

[30] Martín-Alfonso J.E., Franco J.M. (2014). Ethylene-vinyl acetate copolymer (EVA)/sunflower vegetable oil polymer gels: Influence of vinyl acetate content. Polym. Test..

[31] Sánchez M.C., Franco J.M., Valencia C., Gallegos C., Urquiola F., Urchegui R. (2011). Atomic force microscopy and thermo-rheological characterisation of lubricating greases. Tribol. Lett..

[32] Wu S. (1989). Chain structure and entanglement. J. Polym. Sci. B Polym. Phys..

[33] Ferry J.D. (1980). Viscoelastic Properties of Polymers.

[34] Yalcin H., Toker O.S., Dogan M. (2012). Effect of oil type and fatty acid composition on dynamic and steady shear rheology of vegetable oils. J. Oleo Sci..

[35] Yeong S.K., Luckham P.F., Tadros T.F. (2004). Steady flow and viscoelastic properties of lubricating grease containing various thickener concentrations. J. Colloid Interface Sci..

[36] Nayak S.K., Mohanty S. (2009). Dynamic mechanical, rheological, and thermal properties of intercalated polystyrene/organomontmorillonite nanocomposites: Effect of clay modification on the mechanical and morphological behaviors. J. Appl. Polym. Sci..

[37] Yoon J.T., Jo W.H., Lee M.S., Ko M.B. (2001). Effects of comonomers and shear on the melt intercalation of styrenics/clay nanocomposites. Polymer.

[38] Ortega F.J., Navarro F.J., García-Morales M., McNally T. (2015). Thermo-mechanical behaviour and structure of novel bitumen/nanoclay/MDI composites. Compos. Part B Eng..

[39] NLGI (2006). Lubricating Greases Guide.

[40] Gow G., Mortier R.M., FoxStefan M.F., Orszulik T. (2010). Lubricating Grease in Chemistry and Technology of Lubricants.

[41] Brandão J.A., Meheux M., Ville F., Seabra J.H.O., Castro J. (2012). Comparative overview of five gear oils in mixed and boundary film lubrication. Tribol. Int..

[42] Martín-Alfonso J.E., Valencia C. (2015). Tribological, rheological, and microstructural characterization of oleogels based on EVA copolymer and vegetables oils for lubricant applications. Tribol. Int..

[43] Zhang T., Guan E., Yang Y., Liu F., Zhang L., Pang J., Bian K. (2021). Fatty acid profiles of vegetable oils from four different plant sources and their effects on dough rheology and chinese steamed bread quality. Int. J. Food Sci. Technol..

[44] (1991). Standard Test Methods for Cone Penetration of Lubricating Grease. Annual Book of ASTM Standards.

